# A Rare Case of Spontaneous Steinstrasse

**DOI:** 10.7759/cureus.49641

**Published:** 2023-11-29

**Authors:** Luís Cesar Fava Spessoto, Rafael S Aguiar, Guilherme C Gonzales, Ana Clara N Spessoto, Fernando Nestor Facio

**Affiliations:** 1 Urology, Faculty of Medicine of São José do Rio Preto (FAMERP), São José do Rio Preto, BRA; 2 Medicine, Medical School of Catanduva, Catanduva, BRA

**Keywords:** shock wave lithotripsy, urology, treatment, clinic, steinstrasse

## Abstract

Spontaneous steinstrasse (“stone street”) is a collection of stones within the ureter and is a rare and understudied event. Factors such as infection, altered kidney function, and degree of obstruction are used to define the most adequate therapeutic option. Treatment can be either conservative or surgical. The decision of which depends on the clinical presentation. This paper reports a rare case of a 59-year-old patient with spontaneous steinstrasse examined at a urology clinic. Surgical intervention was required because of altered kidney function. The patient is currently undergoing follow-up for the metabolic investigation.

## Introduction

Steinstrasse or “stone street” is an aggregation of particles in the ureter. On x-ray, such collections have the appearance of a cobbled street, hence the term steinstrasse, which means "street of stone" in German. Steinstrasse occurs in up to 15% of cases after extracorporeal shockwave lithotripsy (ESWL) [1], and 6% of these cases require intervention [2]. The incidence is related to factors such as the size of the calculi, location [3], and the energy imposed during ESWL [4]. The main complication of this event is ureteral obstruction, which can occur in up to 23% of cases [5], leading to the loss of kidney function [4].

Post-ESWL steinstrasse is classified into three types. Type 1 is characterized by multiple small fragments. Type 2 has fragments measuring 5 mm or more and small proximal fragments. Type 3 has multiple fragments measuring 5 mm or more [6].

Spontaneous steinstrasse is a spontaneous accumulation of small stones without a preceding surgical intervention, a rare and understudied event [7]. Some factors such as the infection, altered kidney function, and degree of obstruction are used to define the most adequate therapeutic option. Management can be either conservative or surgical. The decision of which depends on the clinical presentation.

This paper presents a rare case of a patient with spontaneous steinstrasse examined at a urology clinic.

## Case presentation

A 59-year-old male patient, hypertensive, visited a urology clinic with the complaint of recurring renal colic on the right side, with no previous urological procedures. Ultrasonography of the kidney and ureters performed two months earlier identified a calculus measuring 1.4 cm in the right ureteropelvic junction and a branched calculus measuring 3.3 cm in the left kidney.

**Figure 1:**
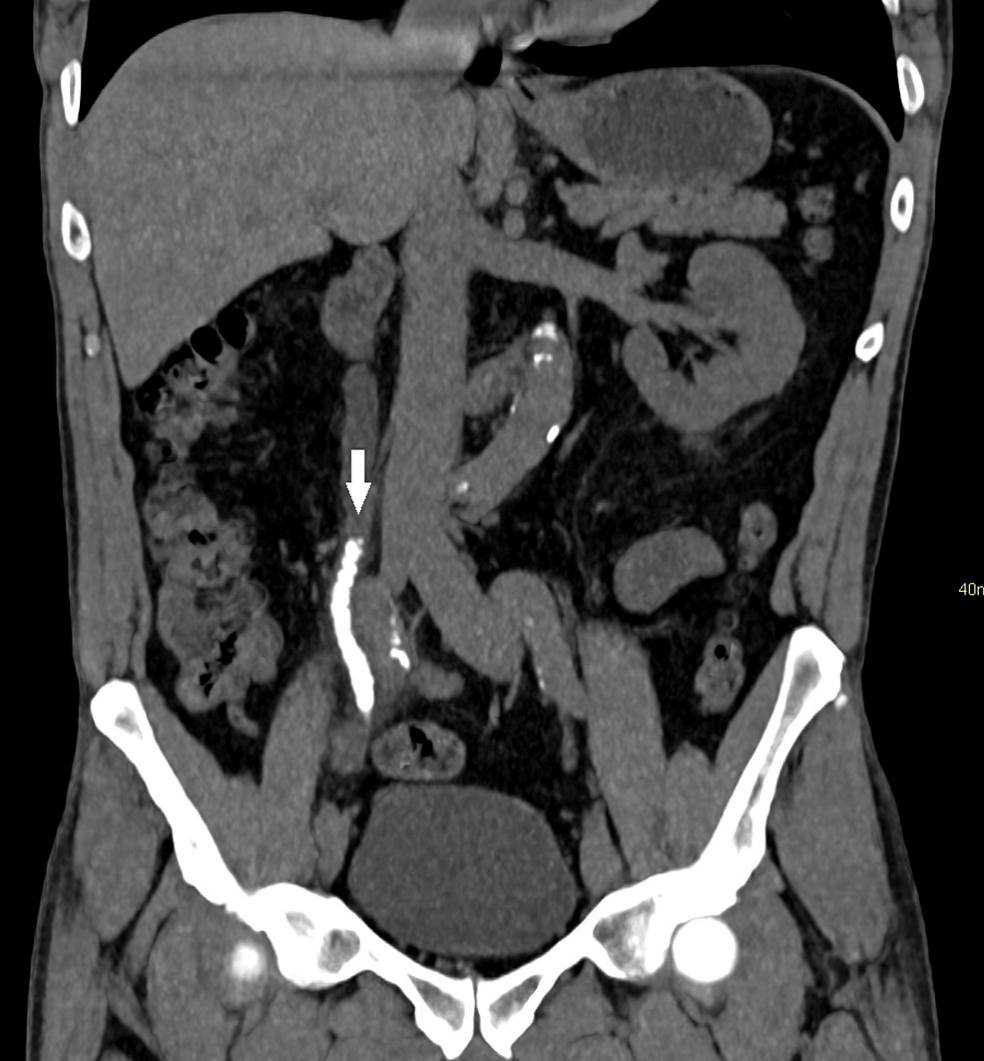
Computed tomography (coronal axis) showing right-side steinstrasse and ipsilateral ureteral dilation (arrow).

The patient was sent to the emergency room. Computed tomography revealed renal lithiasis: calculus measuring 9 mm in the right lower calyx and multiple calculi in the right kidney, with the largest of which being 1.2 cm in the lower calyx; multiple small stones situated along the right ureter (some overlapping), in greater quantity at the crossing of the iliac vessels; and moderate dilation of the right collector system and density of all calculi ranging from 420 to 505 UH (Figure 1).

Laboratory exams revealed kidney function, with serum creatinine of 1.78 mg/dL, urea of 59 mg/dL, and discrete hyperkalemia (5.4 mg/dL), with no associated infection based on the urine exam (Table 1).

**Table 1:** Results of the laboratory exams.

Variables	Admission	Metabolic investigation	Normal range values
Hemoglobin (g/dL)	14.4	-	12.8-16.5
Hematocrit (%)	43.3	-	40.0-54.0
White blood cells (/mm^3^)	9940	-	4.000-11.000
Platelets (/mm^3^)	324000	-	140-450
Creatinine (mg/dL)	1.78	-	0.60-1.20
Urea (mg/dL)	59.50	-	<50
Sodium (mmol/L)	136	141	135-145
Potassium (mmol/L)	5.4	5.3	3.50-5.10
Ionized calcium (mmol/L)	-	1.3	1.10-1.40
Total calcium (mg/dL)	-	9.7	8.6-10.2
Chlorine (mmol/L)	-	106	98-107
Urinary data			
pH	5.0	-	5.0-7.0
Density	1,008	-	1,015-1,025
Nitrite	Negative	-	Negative
White blood cells (/mL)	3,000	-	Up to 25,000
Red blood cells (/mL)	1,000	-	Up to 25,000
Cylinders	Absent	-	Absent
Crystals	Absent	-	Absent
Uroculture	Negative	-	Negative
Venous blood gas			
pH	-	7.36	7.33-7.43
HCO_3 _(mmoL/L)	-	23.9	23-27
BE (mmoL/L)	-	-1.2	-3-3
PO_2_ (mmHg)	-	23	30-50
PCO_2_ (mmHg)	-	43.5	38-50

The patient was submitted to two sessions of ureteroscopy by rigid ureteroscope and laser lithotripter, with a six-week interval because of the stone burden, without complications, resulting in the complete resolution of the ureteral calculi. The patient is currently undergoing follow-up at a nephrology clinic for metabolic investigation of the calculi.

## Discussion

Cases of spontaneous steinstrasse are rare, and different factors contribute to the indication of the best therapeutic option to adopt. In the present case, the patient had type 3 steinstrasse and altered kidney function.

Treatment for this condition can be conservative or surgical, and the decision is directly related to the clinical presentation. In the present case, surgical intervention was performed because of the altered kidney function.

The literature describes the association between spontaneous steinstrasse and nephrocalcinosis with renal tubular acidosis [8]. In the present case, the patient had bilateral nephrolithiasis but no indication of tubular acidosis or nephrocalcinosis. Currently, the patient remains in metabolic investigation and urological follow-up because of the nephrolithiasis.

Analyzing 958 patients with renal stones who underwent ESWL, Kim et al. verified that 63.6% of cases have spontaneous resolution [9]. However, the therapeutic approach to patients with spontaneous steinstrass requires more clinical studies, as the rarity of cases makes the standardization of conduct difficult.

Although conservative conduct is a therapeutic option, patients with persistent symptoms and ureteral obstruction are preferably treated surgically, as in the present case. Thus, when conservative treatment (spontaneous elimination of calculi) is not satisfactory, the conduct should include temporary urinary deviation for the monitoring of infection. With the resolution of this condition, definitive treatment is instituted: surgical removal of the steinstrasse.

## Conclusions

Spontaneous steinstrasse is an uncommon event, for which the therapeutic approach lacks scientific evidence. The most adequate therapeutic option depends on the patient’s clinical condition and the size of the calculi. Patients with persistent symptoms and ureteral obstruction are preferably treated surgically.
